# Vector Competence of Florida *Culicoides insignis* (Diptera: Ceratopogonidae) for Epizootic Hemorrhagic Disease Virus Serotype-2

**DOI:** 10.3390/v13030410

**Published:** 2021-03-05

**Authors:** Bethany L. McGregor, Dinesh Erram, Barry W. Alto, John A. Lednicky, Samantha M. Wisely, Nathan D. Burkett-Cadena

**Affiliations:** 1 Center for Grain and Animal Health Research, Agricultural Research Service, United States Department of Agriculture, Manhattan, KS 66502, USA; 2 Florida Medical Entomology Laboratory, Institute of Food and Agricultural Sciences, University of Florida, Vero Beach, FL 32962, USA; derram@ufl.edu (D.E.); bwalto@ufl.edu (B.W.A.); nburkettcadena@ufl.edu (N.D.B.-C.); 3 Department of Environmental & Global Health, College of Public Health and Health Professions, University of Florida, Gainesville, FL 32610, USA; jlednicky@phhp.ufl.edu; 4 Department of Wildlife Ecology and Conservation, Institute of Food and Agricultural Sciences, University of Florida, Gainesville, FL 32611, USA; wisely@ufl.edu

**Keywords:** arboviruses, *Reoviridae*, *Orbivirus*

## Abstract

Epizootic hemorrhagic disease virus (EHDV; family *Reoviridae*, genus *Orbivirus*) is an arthropod-borne virus of ungulates, primarily white-tailed deer in North America. *Culicoides sonorensis*, the only confirmed North American vector of EHDV, is rarely collected from Florida despite annual virus outbreaks. *Culicoides insignis* is an abundant species in Florida and is also a confirmed vector of the closely related Bluetongue virus. In this study, oral challenge of *C. insignis* was performed to determine vector competence for EHDV serotype-2. Field-collected female midges were provided bovine blood spiked with three different titers of EHDV-2 (5.05, 4.00, or 2.94 log_10_PFUe/mL). After an incubation period of 10 days or after death, bodies and legs were collected. Saliva was collected daily from all females from 3 days post feeding until their death using honey card assays. All samples were tested for EHDV RNA using RT-qPCR. Our results suggest that *C. insignis* is a weakly competent vector of EHDV-2 that can support a transmissible infection when it ingests a high virus titer (29% of midges had virus positive saliva when infected at 5.05 log_10_PFUe/mL), but not lower virus titers. Nevertheless, due to the high density of this species, particularly in peninsular Florida, it is likely that *C. insignis* plays a role in the transmission of EHDV-2.

## 1. Introduction

Epizootic hemorrhagic disease virus (EHDV) is an arthropod-borne virus, transmitted by biting midges of the genus *Culicoides* (Diptera: Ceratopogonidae), that causes disease in ungulates of the families Cervidae and Bovidae [1]. Seven serotypes of EHDV exist worldwide, with three serotypes currently present in the United States (EHDV-1, EHDV-2, and EHDV-6) [2]. Infection with EHDV can result in extreme morbidity and mortality of infected animals, primarily white-tailed deer (*Odocoileus virginianus*) in North America, resulting in significant impacts to wild populations and devastating losses for North American deer farmers [2,3]. These pathogens can also impact other economically important animal industries, such as cattle operations, resulting in significant losses to milk production in dairy cattle [4].

Currently, *Culicoides sonorensis* Wirth and Jones is the only confirmed vector of EHDV in North America [5,6] and is competent for the three endemic North American EHDV serotypes: EHDV-1, EHDV-2, and EHDV-6 [7,8], as well as for the exotic serotype EHDV-7 [9]. The range of *C. sonorensis* in the United States is primarily west of the Mississippi River [10], although sporadic populations do occur in the eastern half of the country [10,11], and increasing northeastern range expansion has been documented [12]. Despite periodic EHDV outbreaks in the southeastern US, multiple large-scale collection efforts have recorded few to no *C. sonorensis* [13,14,15,16], suggesting the role of other species in the transmission of EHDV in this region.

The *Culicoides* fauna of North America is diverse, representing around 150 species [10]. A subset of these species stands out as potential vector candidates due to their relative abundance during EHDV outbreaks, as well as their propensity to feed heavily on susceptible ruminant hosts. This group includes species such as *C. biguttatus* (Coquillett), *C. debilipalpis* Lutz, *C. obsoletus* Meigen, *C. pallidicornis* Kieffer, *C. paraensis* Goeldi, *C. spinosus* Root and Hoffman, *C. stellifer* (Coquillett), and *C. venustus* Hoffman [13,14,17,18,19,20]. Despite the identification of many potential vector candidate species, the small size of *Culicoides* and challenges to their colonization have largely limited the ability of researchers to evaluate their vector competence.

Another North American EHDV vector candidate is *Culicoides insignis* Lutz, often a highly abundant species near susceptible animals, including cattle and white-tailed deer, particularly in peninsular Florida [16,21,22]. Considered a tropical species, *C. insignis* has historically been reported mostly in the southernmost continental US state of Florida, with seasonal incursions into Georgia and Alabama [10]. However, recent findings indicate that this species is currently exhibiting a northwestern range expansion into Mississippi and Louisiana [23]. This distribution reflects regions for which recurrent outbreaks of EHDV have occurred [13,24], but where *C. sonorensis* has frequently been absent from collections [13,14,15,16]. *Culicoides insignis* is also a confirmed vector of Bluetongue virus (BTV) [25], another domestic ungulate-affecting *Orbivirus*, with close genomic similarity to EHDV [26]. The close antigenic relationship between these two viruses led to early postulation that *Culicoides*, the known vector genus for BTV, might also be the vector of EHDV [27]. Multiple studies have also shown that *Culicoides* species that are competent for one virus are often competent for the other, a phenomenon seen with *C. bolitinos* Meiswinkel, *C. brevitarsis* Kieffer, *C. imicola* Kieffer and *C. sonorensis* [6,28,29,30,31,32]. For the reasons discussed, *C. insignis* is a suspected EHDV vector candidate. The goal of the present study was to determine the vector potential of field-collected *C. insignis* for EHDV-2 through vector competence assays.

## 2. Materials and Methods

### 2.1. Field Collections of Culicoides insignis

**Figure 1:**
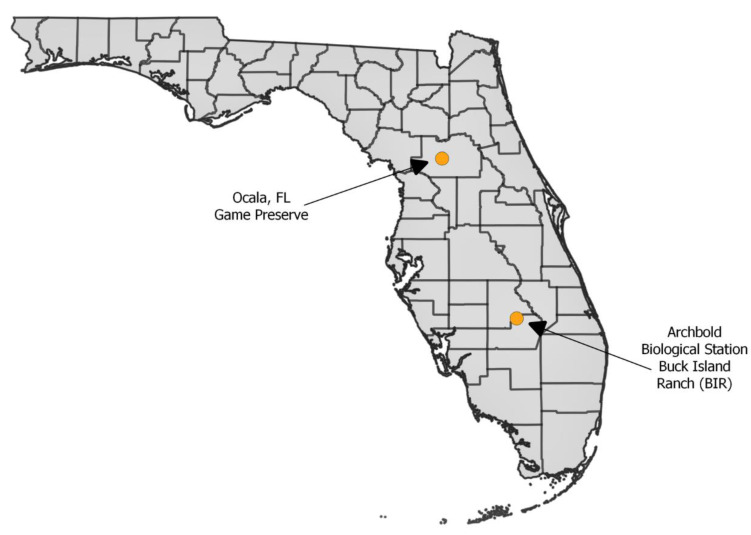
Location of the two field sites in Florida from where *Culicoides insignis* midges were collected. Map generated using QGIS3 and shapefiles from the Florida Geographic Data Library database.

*Culicoides insignis* females used for this study were collected from two sites in the Florida peninsula. The two sites were: (a) Archbold Biological Station’s Buck Island Ranch (BIR), which is located south of Lake Placid (Highlands County) and is a research rangeland for investigating the relationships between cattle ranching, citrus production and Florida native ecosystems, and (b) a privately owned deer farm near Ocala (Marion County) (Figure 1). Biting midges were collected using Centers for Disease Control and Prevention (CDC) miniature light traps (Model 2836BQ, BioQuip Inc., Rancho Dominguez, CA, USA) with LED black light arrays (model 2790V390, BioQuip Inc.) that were baited with CO_2_ (dry ice). The traps were modified with mesh (standard nylon window screen) at the intake to exclude larger arthropods. A BugDorm insect cage (Model #4F2222, BugDorm, Taiwan) was used as the collection net to increase survival of trapped insects [33]. Due to low blood-feeding rates, multiple collections had to be made for each infection trial. Collections were made on select nights between 11 and 17 July 2018 at the BIR site and between 12 and 26 September 2018 at the Ocala site. Traps were retrieved in the morning and returned to the Florida Medical Entomology Laboratory where midges were aspirated into 16oz paperboard cups and placed into an incubator (Thermo Fisher Scientific, Waltham, MA, USA) at 25 °C, 80% humidity, and 14:10 light:dark (L:D) photoperiod. Prior to blood-feeding, which was conducted within 2 h of arrival at the laboratory, midges were only provided with water.

### 2.2. Viral Screening of Field-Collected Midges

Field-collections for these studies took place on farms reporting no EHDV related mortality at the time of collection. In order to determine whether EHDV-2 was naturally circulating in the populations used for this study, any midges not used for the experimental infection trials were tested for the presence of EHDV-2 RNA by pool screening. None of the midges included in viral screening had any observable blood in their gut. *Culicoides insignis* midges were sorted into pools of 50 whole individuals in 1.5 mL microcentrifuge tubes containing 500 µL medium 199 (HyClone Medium 199, GE Healthcare Life Sciences, Logan, UT, USA) with 10–20 2 mm borosilicate beads (Sigma-Aldrich, St. Louis, MO, USA) for homogenization, which was conducted using the Bullet Blender Storm (Next Advance, Troy, NY, USA) for five minutes following manufacturer’s protocols. Samples were then processed using the QiaAmp Viral RNA Mini Kit (Cat#52906, Qiagen, Hilden, Germany) following manufacturer’s protocols for extraction of viral RNA.

Following RNA extraction, RT-qPCR was conducted using the SuperScript III One-Step qRT-PCR kit (Thermo Fisher Scientific) following established protocols [34]. Primer and probe sequences from Wernike et al. [35] were used and reaction conditions were modified from Wilson et al. [36] to 25 min at 55 °C, 2 min at 95 °C, and 45 cycles of 10 s at 95 °C and 1 min at 57 °C. Positive and negative controls were used on all RT-qPCR assays.

### 2.3. Per Os Infections of Culicoides

The EHDV-2 strain used for the infection study was isolated by the University of Florida’s Cervidae Health Research Initiative in 2016 from the spleen of an infected white-tailed deer in Gadsden County, Florida (GenBank Accession Numbers MF688816.1–MF688825.1). The virus was passaged twice in Vero cells (African green monkey kidney cells), originating from American Type Culture Collection (ATCC, Manassas, VA, USA), prior to use. Cells were cultured in medium 199 with Earle’s Balanced Salt Solution complete with 10% fetal bovine serum (Atlanta Biologicals, Flowery Branch, GA, USA), 2% penicillin streptomycin solution (Gibco, Thermo Fisher Scientific, Waltham, MA, USA), and 0.2% Amphotericin B solution (Gibco, Thermo Fisher Scientific). Standard curves were generated through plaque assays and RT-qPCR of serially diluted virus samples for infectious titer determination as plaque forming unit equivalents per milliliter (PFUe/mL).

A blood-feeding apparatus was developed based on the designs of Venter et al. [37] using readily available laboratory materials. Infectious bloodmeals for oral challenge of midges to EHDV-2 were prepared by spiking defibrinated bovine blood (HemoStat Laboratories, Dixon, CA, USA) with fresh virus grown immediately preceding the bloodmeal. Infection trials were conducted using different virus concentrations including 5.05 log_10_PFUe/mL (high titer), 4.00 log_10_PFUe/mL (medium titer), and 2.94 log_10_PFUe/mL (low titer) EHDV-2. For each feeding trial, blood samples were taken prior to and after each feed to determine infectious titers. Blood feeding took place inside an incubator at 25 °C and 80% humidity in dark conditions to replicate the crepuscular and nocturnal environment in which midges typically feed. Subsequently, midges were anesthetized using 10 µL triethylamine (TEA) (Fisher Scientific 04884-100, Atlanta, GA, USA) diluted 10x in ethanol applied to a cotton pad. Midges were exposed to the TEA solution until the last midge ceased moving, typically 2–3 min [33]. Fully engorged *C. insignis* midges were identified to species using morphological characteristics [38] and unfed midges were discarded. Midges were not identified prior to feeding to limit the number of times individuals had to be anesthetized, which could compromise survival. Individual females were then placed into 4oz paperboard cups covered with no-see-um netting and provided 10% sucrose solution for the first two days and switched to honey cards from day 3 onwards. Cups were placed in an incubator maintained at 25 °C, 80% humidity, and a 14:10 L:D photoperiod.

### 2.4. Midge Tissue Collection and Processing

Midges were held for an incubation period of up to 10 days [39] after blood feeding at which point all midges were anesthetized using a CO_2_ pad (Item #BGSU-12, Lab Scientific, Danvers, MA, USA) for processing. Legs and wings were dissected and collected into 1.5 mL microcentrifuge tubes. The body of the midge was placed into a separate 1.5 mL tube. Both tubes contained 150 µL medium 199 and 5–10 2 mm borosilicate glass beads. For midges that died between 3 and 10 days post-feed (dpf), leg/wing and body samples were collected within 12 h of death.

To gauge transmission potential, saliva was collected using honey card assays [34] from 3 dpf onwards. For these assays, a small piece of filter paper (Whatman grade 1 filter paper, cut to <1 cm^2^) was coated in honey and placed on the mesh of each study cup as the sole sugar source for the midges. Each day, the filter paper was collected into a 1.5 mL microcentrifuge tube containing 150 µL medium 199 and a new honey-coated filter paper (honey card) was given to the females.

Body and leg samples were homogenized using the Bullet Blender Storm for five minutes following manufacturer’s protocols. Honey cards were ground in 150 µL medium 199 using a pestle to initiate release of virus from filter paper. Protocols for RNA extraction and RT-qPCR were identical to those described above for virus detection in field collected midges and all RT-qPCR assays included positive and negative controls.

### 2.5. Statistical Analysis

In total, three *per os* infection trials were used to investigate *C. insignis* EHDV-2 vector competence following oral ingestion of blood with concentrations of infectious virus. Infection, dissemination, and transmission potential rates were calculated separately for each trial. Infection rates were calculated by dividing the total number of individuals with infected bodies by the total number of individuals that originally blood fed. Dissemination, the process whereby virus emerges from the midgut of the insect and moves into other body tissues, was tested with the legs and wings. Dissemination rates were calculated by dividing the total number of individuals with positive legs and wings over total individuals with positive bodies. Finally, transmission potential was calculated by dividing the total number with virus-positive saliva by the total number of individuals with virus-positive bodies [40,41]. Overall rates of infection, dissemination, and transmission potential for each trial were also calculated by dividing the number with positive bodies, legs/wings, and saliva, respectively, by the total number of individuals that ingested an infectious blood meal.

Infection, dissemination, and transmission rates were compared between infection titers statistically using Fisher’s exact tests to investigate whether infectious titer significantly impacted the course of infection within the midge cohorts. When significance was found through Fisher’s exact tests, pairwise comparisons using a Bonferroni correction were used to further investigate significant differences. Viral titers were evaluated to determine whether they followed a normal distribution using a Shapiro–Wilk test followed by analysis with a one-way ANOVA where adequate data were available. All statistical analyses were run using the *stats* package in R software version 3.3.3 [42] and evaluated at α = 0.05.

### 2.6. Intrathoracic Inoculation Assays

In order to determine whether a salivary gland infection and escape barrier exists in these insects, a subset of individuals collected from the BIR site were intrathoracically injected with EHDV-2 to bypass midgut infection and escape barriers. Midges were anesthetized using a CO_2_ pad and injected with 45 nL of viral media at 5.0 log_10_PFUe/mL using the Nanoject II auto-nanoliter injector (Drummond Scientific, Broomall, PA, USA) fitted with microinjection needles pulled from 9 cm long glass capillary tubes (Drummond Scientific, Broomall, PA, USA). Needles were changed after every five individuals to maintain a sharp point capable of piercing the insect’s cuticle cleanly. After injection, midges were placed into individual 4oz cups covered with no-see-um mesh and provided with 10% sucrose solution. Microinjected midges were then monitored for 48-h post injection to detect injection-related mortality, which is typically expected within 24 h of the inoculation [43]. For survivors, honey cards were administered daily from 2 days post-injection until their death. Whole midge bodies and honey cards were processed as described above and viral titers were determined based on viral standards.

## 3. Results

### 3.1. Viral Screening of Pooled Field-Collected Midges

Screening was conducted on 17,800 wild *C. insignis* females in 356 pools of 50 whole individuals each. Samples were screened for viral RNA through RT-qPCR and no positive pools were detected. It is unlikely that EHDV was naturally circulating in the populations of midges used in the infection trials.

### 3.2. Infection, Dissemination, and Transmission Potential of C. insignis

**Figure 2:**
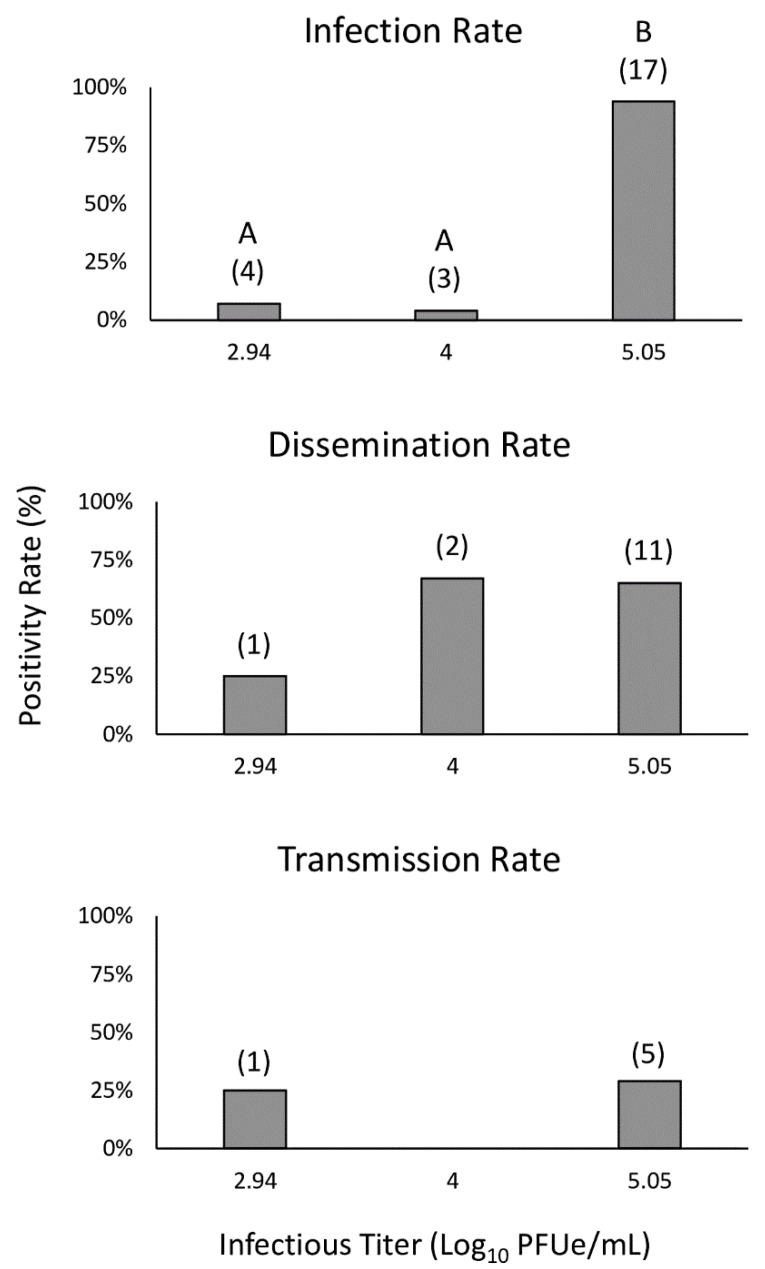
Infection, dissemination, and transmission rates for *per os* infection trials conducted with three infectious titers of EHDV-2. Fisher’s exact tests indicated significant differences in infection rates (*p* < 0.001). Pairwise significance is indicated above bars for infection rates. Infection rates were not significantly different between the 2.94 and 4.00 log_10_PFUe/mL infections (A); however, infection rates were significantly higher when midges were fed an infectious titer of 5.05 log_10_PFUe/mL (B) than when they were fed either of the lower titers tested. No significant difference was found for dissemination rates (*p* = 0.22) or transmission rates (*p* = 0.47), so pairwise significance was not tested. Numbers in parenthesis indicate the number of positive samples.

**Figure 3:**
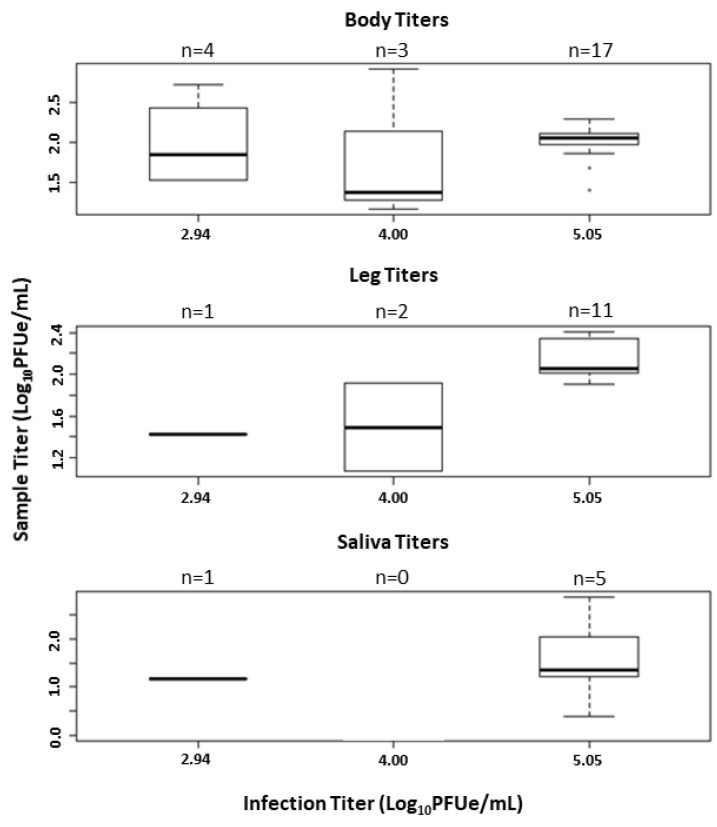
Viral titers of EHDV-2 in bodies, legs, and saliva of *Culicoides insignis* infected at three viral titers. Center lines of boxplots represent the median viral titer. Upper and lower extent of boxes represent the third and first quartile, respectively. Whiskers represent the estimated maximum and minimum values (1.5× the interquartile length). Points outside of whiskers represent outliers. Plots represented by a solid horizontal line represent one data point for that treatment. No significant difference in viral titer was detected between treatments for body titers (*p* = 0.30). Sample sizes for leg and saliva titers were low, excluding the possibility of meaningful statistical comparison.

The results of three *C. insignis* vector competence trials are provided in Table 1. In trial one, midges from the BIR population were fed blood containing a moderately high viral titer of 5.05 log_10_PFUe/mL of EHDV-2. Only 18 midges fully engorged on blood during this first trial. The infection rate for trial one was high, with positive bodies from 17/18 individuals (94.4%, Figure 2) at a mean viral titer (±SE) of 2.00 ± 0.05 log_10_PFUe/mL (Figure 3). Dissemination rates remained high during this trial at 11/17 individuals (64.7%, Figure 2) with a mean viral titer of peripheral tissues at 2.14 ± 0.06 log_10_PFUe/mL (Figure 3). Transmission potential for trial one was 29.4% (Figure 2) with 5/17 individuals having positive saliva at a mean viral titer of 1.58 ± 0.42 log_10_PFUe/mL (Figure 3; Supplemental File S1).

In trial two, midges from the Ocala population were fed a titer of 4.00 log_10_PFUe/mL EHDV-2, with 70 individuals becoming engorged. Three of these 70 individuals (4.3%, Figure 2) developed an infection in body tissues at just 1.82 ± 0.55 log_10_PFUe/mL (Figure 3). Two of these three developed disseminated infections (Figure 2) with a mean viral titer of 1.49 ± 0.42 log_10_PFUe/mL (Figure 3). Neither midge developed detectable virus in salivary samples (Figure 2).

For the final trial, Ocala population midges were fed a low viral titer of 2.94 log_10_PFUe/mL of EHDV-2, resulting in 54 blood engorged individuals. Positive bodies were detected for 4/54 individuals (7.4%, Figure 2) with a detected viral titer of 1.98 ± 0.28 log_10_PFUe/mL (Figure 3). Only one of these four individuals presented with positive peripheral tissues (25.0%, Figure 2) at a viral titer of 1.43 log_10_PFUe/mL (Figure 3). The same individual had detectable virus in saliva (Figure 2) at a viral titer of 1.17 log_10_PFUe/mL (Figure 3).

### 3.3. Infectious Titer Comparisons

There was a significant difference in the infection rate of midges fed bloodmeals containing different viral titers (*p* < 0.001, Figure 2). Pairwise comparisons found that the infection rates were significantly different between midges fed a high titer and medium titer blood meal (Fisher’s Exact *p* < 0.001) and a high titer and low titer blood meal (Fisher’s Exact *p* < 0.001), but not between a medium and low titer blood meal (Fisher’s Exact *p* = 1). The Fisher’s exact tests for dissemination rate (*p* = 0.22) and transmission rate (*p* = 0.47) were not significant based on infectious titer, so no further pairwise comparisons were made.

Viral titer data for body samples were determined to be normally distributed (*p* = 0.19), permitting the use of one-way ANOVA. ANOVA results indicated no significant difference in viral titer in body samples between infection titers (F_1,22_ = 0.07, *p* = 0.79). Leg and saliva titers were not compared between infection titers due to low dissemination and transmission rates in medium and low titer trials.

### 3.4. Intrathoracic Inoculation Assays

Microinjection was conducted on 28 midges, with 12 individuals surviving the first 48 h to the start of honey card monitoring for transmission. Altogether, five of these 12 individuals developed detectable virus in saliva (41.7%). Two of these five (40.0%) had detectable virus in saliva by two days post injection. The other three developed detectable virus in saliva by three days post injection.

## 4. Discussion

This is the first study characterizing vector competence and associated virus barriers of field collected *C. insignis* for EHDV-2. Furthermore, the strain used represented a recently isolated strain present in Peninsular Florida within the known range of *C. insignis.* Based on the results of the current study, *C. insignis* is a weakly competent vector for EHDV-2 capable of developing infection, dissemination, and transmission potential when feeding on blood containing a viral titer of 5.05 log_10_PFUe/mL. However, infection waned when midges were fed blood containing lower viral titers indicating dose-dependent barriers to infection.

There are four barriers to transmission that are commonly associated with vector-borne disease [44]. These include the midgut infection barrier, midgut escape barrier, salivary gland infection barrier, and salivary gland escape barrier. Previous evidence shows variation in these barriers in *Culicoides* borne pathogens with *C. sonorensis* demonstrating a midgut infection and escape barrier but lacking a salivary gland infection and escape barrier for BTV [45]. For EHDV, midgut infection and escape barriers appear to be weak in *C. sonorensis*, with a moderate salivary gland barrier in place [39]. The present study adds to our knowledge of these barriers in *Culicoides.* In trial 1, high infection and dissemination rates indicate that midgut infection and escape barriers are overcome in *C. insignis* at oral infection titers of 5.05 log_10_PFUe/mL EHDV-2 and presumably higher. This viral titer is slightly higher than some detected viremias in EHDV-2 experimentally infected deer [46], although comparable to viral titers of experimentally infected deer exposed to the closely related EHDV-7 [47]. However, transmission potential during *per os* infection trials and intrathoracic inoculation assays was between 29 and 42%, indicating the presence of salivary gland infection and escape barriers for *C. insignis.* In trials 2 and 3 of this experiment, very low infection rates were recorded, indicating that at infectious titers of 3–4 log_10_PFUe/mL, the virus was unable to establish a sufficient midgut infection to promote further infection of body tissues.

When compared with *C. sonorensis*, the detected competence of *C. insignis* in the present study is much lower, especially as it pertains to dissemination and transmission potential. Some cohorts from a previous study of *C. sonorensis* exposed to the same strain of EHDV-2 at 5.5 log_10_PFUe/mL displayed up to 100% infection, 100% dissemination, and 79% transmission by 10 days post infection [34]. Although, it is important to note that these values are high compared with results from other studies using different EHDV-2 strains, infection titers, and detection methods. For example, in one study, infection rates of *C. sonorensis* fed 3.92 log_10_TCID_50_/mL peaked at 47.1% when detected using cellular assays [46]. Another study identified up to 65% infection by cellular assays and up to 90% infection by molecular assays at an initial infection titer of 6.9 log_10_PFU/mL [48]. Infection titer and strain play a significant role in the epidemiology of EHDV-2 and detection methods have different levels of sensitivity. All of these factors should be considered when comparing the vector potential of different species of *Culicoides* [34].

Due to their small size and difficulty in colonizing most species, very few *Culicoides* species have been fully assessed for vector competence in controlled laboratory studies. One successful infection study of *C. debilipalpis* (as *C. lahillei*) with EHDV-2 identified low rates of infection after feeding on blood containing high titers of the virus (5.3–6.0 log_10_TCID_50_/mL) [49]. A population of *C. venustus* from New York also showed low infection rates when exposed to EHDV-1 in a laboratory [17]. However, both of these studies investigated only midge infection rates and did not assess the transmission potential through the presence of EHDV in saliva expectorate, thereby not determining the full vector competence of these species. In the present study, infection and transmission were both assessed in *C. insignis* to fully evaluate vector competence for this species. However, collecting saliva from *Culicoides* is challenging and there is evidence to indicate that false negatives are common with honey card salivary assays [34]. Additionally, it is unclear whether additional individuals would develop a salivary gland infection or whether salivary titers would increase with additional incubation time. Evidence from BTV studies indicates that 14 days of extrinsic incubation may be necessary for successful transmission to occur [25,50]. The shorter incubation period in the present study may have artificially lowered the detectable virus that would be present if a longer incubation period were possible. Due to these factors, our estimate for transmission potential is conservative. Unfortunately, mortality of field-collected midges in captivity was high and longer incubation periods as well as additional saliva collection methods were not tractable in the present study.

The use of field specimens for an infection study is not ideal because of the inability to ascertain factors such as age, nutrition, and impacts of other conditions of the individuals used. However, due to challenges with laboratory colonization, the use of field-collected specimens for vector competence trials, particularly for *Culicoides* species, is somewhat standard [21,26,51,52]. To determine the likelihood that midges used for these trials were not previously infected with EHDV in the field, pool testing of midges was conducted. No known EHDV related outbreaks occurred on the sites where *C. insignis* were collected during this study, indicating that if transmission happened, it would have been at very low levels. *Culicoides insignis* is very abundant throughout most of its range [16,21,53], so if EHDV were present in the population at low levels, significant screening would be needed to detect EHDV-positive individuals. After testing almost 18,000 field-collected *C. insignis* individuals, the lack of any EHDV positive pools indicates an exceptionally low probability that field collected midges used in this study would have been naturally infected.

One important consideration for this study was the collection of *C. insignis* from two separate populations in Florida. Local midge abundance can be highly variable, leading to the need to sample midges from two populations to complete all aspects of this study. For this reason, it is challenging to parse out how much variation is attributable to the use of different populations. These two populations were separated by around 250 km distance and could vary in their vector competence for EHDV. Studies from other *Culicoides*-borne pathogens have found significant population level variation in vector competence for BTV [54] and African horse sickness virus [52], both of which are in the same genus as EHDV, *Orbivirus*. This highlights the importance of investigating differences between *Culicoides* populations in more detail to gain a better understanding of the role population plays in determining vector competence of this species for EHDV.

This study reports findings on the vector competence of *C. insignis*, a common species throughout peninsular Florida and an increasingly common species in the southeastern United States, for EHDV-2. Our results demonstrate that this species is capable of supporting infection, dissemination, and transmission of this pathogen when infected at high titers of EHDV-2, but that competence decreases sharply at lower infection titers. These findings indicate that *C. insignis* may be a weakly competent vector species within its range. Recent evidence from northern Florida indicates that multiple species are likely contributing to the transmission of EHDV-2 in that region [55], reinforcing the idea that EHDV transmission dynamics are complex and likely involve multiple vector species with variable vectorial capacities to support outbreaks.

## Figures and Tables

**Table 1:** Infection, dissemination, and transmission rates of *Culicoides insignis* orally infected with EHDV-2 in trials 1, 2, and 3 as well as microinjected midges (MI). Titers shown in log_10_PFU equivalents/mL and numbers in parentheses show the number of samples positive from total tested. Only the transmission rate is shown for the microinjected trial since injection bypasses midgut infection and escape barriers.

				% Overall Rates			% Adjusted Rates	
Trial	Population	Titer	N	IR ^a^	DR ^a^	TR ^a^	DR ^b^	TR ^b^
1	Buck Island Ranch	5.05	18	94.4 (17)	61.1 (11)	27.8 (5)	64.7 (11)	29.4 (5)
2	Ocala	4.00	70	4.3 (3)	2.9 (2)	0.0 (0)	66.7 (2)	0.0 (0)
3	Ocala	2.94	54	7.4 (4)	1.9 (1)	1.9 (1)	25.0 (1)	25.0 (1)
MI	Buck Island Ranch	5.00	12	-	-	41.7 (5)	-	-

^a^ Denominators for overall rates were the total number of *C. insignis* individuals tested (N). ^b^ Denominators for adjusted rates were the number of positive midge bodies in the IR column.

## Data Availability

Data are available as Supplemental File S1.

## Supplementary Materials

The following are available online at https://www.mdpi.com/1999-4915/13/3/410/s1, Supplemental File S1. Experimental Data.
